# Serum uric acid level, blood pressure, and vascular angiotensin II responsiveness in healthy men and women

**DOI:** 10.14814/phy2.12235

**Published:** 2014-12-11

**Authors:** Arian Samimi, Sharanya Ramesh, Tanvir C. Turin, Jennifer M. MacRae, Magdalena A. Sarna, Raylene A. Reimer, Brenda R. Hemmelgarn, Darlene Y. Sola, Sofia B. Ahmed

**Affiliations:** 1 Faculty of Medicine, University of Alberta, Calgary, Alberta, Canada; 2 Libin Cardiovascular Institute of Alberta, CalgaryAlberta, Canada; 3 Faculty of Medicine, University of Calgary, Calgary, Alberta, Canada; 4 Alberta Kidney Disease Network, Calgary, Alberta, Canada; 5 Department of Biochemistry and Molecular Biology, University of Calgary, Calgary, Alberta, Canada; 6 Faculty of Kinesiology, University of Calgary, Calgary, Alberta, Canada

**Keywords:** Blood pressure, hypertension, renin–angiotensin system, sex, uric acid

## Abstract

Uric acid is associated with hypertension and increased renin–angiotensin system activity, although this relationship diminishes after chronic exposure to high levels. Uric acid is more strongly associated with poor outcomes in women compared to men, although whether this is due to a sex‐specific uric acid‐mediated pathophysiology or reflects sex differences in baseline uric acid levels remains unknown. We examined the association between uric acid and vascular measures at baseline and in response to angiotensin‐II challenge in young healthy humans. Fifty‐two subjects (17 men, 35 premenopausal women) were studied in high‐salt balance. Serum uric acid levels were significantly higher in men compared to women (328 ± 14 μmol/L vs. 248 ± 10 μmol/L, *P* < 0.001), although all values were within normal sex‐specific range. Men demonstrated no association between uric acid and blood pressure, either at baseline or in response to angiotensin‐II. In stark contrast, a significant association was observed between uric acid and blood pressure at baseline (systolic blood pressure, *P* = 0.005; diastolic blood pressure, *P* = 0.02) and in response to angiotensin‐II (systolic blood pressure, *P* = 0.035; diastolic blood pressure, *P* = 0.056) in women. However, this sex difference lost significance after adjustment for baseline uric acid. When all subjects were stratified according to high (>300 μmol/L) or low (≤300 μmol/L) uric acid levels, only the low uric acid group showed a positive association between uric acid and measures of vascular tone at baseline and in response to angiotensin‐II. Differences in uric acid‐mediated outcomes between men and women likely reflect differences in exposure to increased uric acid levels, rather than a sex‐specific uric acid‐mediated pathophysiology.

## Abstract (precis)

The lower baseline uric acid levels in women compared to men may account for differences in uric acid‐associated vascular outcomes rather than sex‐mediated differences in pathophysiology.

## Introduction

Serum uric acid (UA) is associated with hypertension and cardiorenal risk (Zoccali and Mallamaci 2013), although UA appears to have different effects on blood pressure (BP) depending on level and length of exposure (Feig et al. 2013). Furthermore, studies have suggested a sex‐dependent influence of UA level, with higher levels demonstrating a stronger association with poor outcomes in women (Kivity et al. 2013a,b; Yoshitomi et al. 2014), although whether this is a reflection of pathophysiological sex differences or differences in baseline UA levels, which are traditionally lower in women (Mikkelsen et al. 1965), is uncertain.

The pathophysiology linking UA and vascular disease is not clear, but animal and human studies have highlighted a potential role for the renin–angiotensin system (RAS) (Mazzali et al. 2001, 2002; Perlstein et al. 2004), activation of which is detrimental to cardiorenal outcomes (Brewster et al. 2003).

As the impact of UA on vascular outcomes has been postulated to be greatest in the younger, healthier population (Feig et al. 2013), we sought to evaluate the association between UA levels and measures of BP and arterial stiffness in a young population free of comorbidities. The aim of our study was to determine if the association between UA and measures of arterial tone is sex dependent or is simply a function of different baseline UA levels. We hypothesized that any association between UA and measures of arterial tone, both at baseline and in response to angiotensin II (Ang‐II), as a marker of increased vascular RAS activity (Shoback et al. 1983a), would be influenced by baseline UA levels in healthy men and women.

## Methods

### Subjects

Fifty‐two healthy (17 men, 35 women), normotensive, nondiabetic subjects were enrolled in the study. All were nonobese, nonsmokers and taking no prescription medications (including oral contraceptives). Subjects underwent a medical history, physical examination, and laboratory screening. The study protocol was approved by the Conjoint Health Research Ethics Board at the University of Calgary. Written informed consent was obtained from all study participants in accordance with the Declaration of Helsinki.

### Protocol

Subjects were instructed to consume >200 mmol sodium/day for 3 days before the study to ensure maximal RAS suppression (Shoback et al. 1983b). A 24‐h urine collection or a 2nd morning spot urine sample (Kawasaki et al. 1993) was used to determine urinary sodium, creatinine, and albumin. Subjects were studied in the supine position in a warm, quiet room after an 8 h fast. All women were studied 14 days after the 1st day of the last menstrual period, determined by counting days and measuring 17β‐estradiol levels. At 8 am, an 18‐gauge peripheral venous cannula was inserted into the antecubital vein of each arm (1 for infusion, 1 for blood sampling). After a 90‐min equilibration period, BP was measured at baseline and in response to two doses of Ang‐II (3 ng × kg^−1^ × min^−1^ × 30 min, 6 ng × kg^−1^× min^−1^ × 30 min) as an index of RAS activity (Shoback et al. 1983a). Blood samples were collected at baseline and every 30 min until the end of the study.

### BP measurements

BP was recorded every 15 min by an automatic recording device (Dinamap; Critikon, Tampa, FL). Subjects were studied in the supine position using a standard cuff placed on the right arm at rest. The mean of two readings taken by the same registered nurse was reported.

### Measurement of vascular tone

Measures of arterial stiffness were determined at baseline and in response to Ang‐II infusion, as outlined above. The aortic augmentation index (AIx) was determined by applanation tonometry of the right radial artery using a Millar piezoresistive pressure transducer (Millar SPT 301; Millar Instruments, Houston, TX) coupled to a Sphygmocor device (PWV Medical).

Pulse‐wave velocity (PWV) was determined by sequential acquisition of pressure waveforms from the carotid to the radial arteries or from the carotid to the femoral arteries by the use of same tonometer. The timing of these waveforms was compared with that of the R‐wave on a simultaneously recorded ECG. PWV_carotid‐radial_ was measured in the first 33 subjects before a protocol change resulting in measurement of PWV_carotid‐femoral_ in the subsequent 19 subjects. No individuals had both their PWV_carotid‐femoral_ and PWV_carotid‐radial_ measured. However, a sensitivity analysis using only carotid‐femoral/carotid‐radial PWV revealed similar results.

### Laboratory measurements

Uric acid was determined by an enzymatic colorimetric assay (Roche c501; Roche, Indianapolis, IN). Urinary sodium was determined by an indirect potentiometry assay using an ion‐selective electrode (Roche Cobras Integra Sodium; Roche). Urinary albumin excretion was determined using an immunoturbidimetric assay for quantification of albumin in human urine (Integra 800; Roche). Cholesterol levels were determined by an enzymatic colorimetric assay (Roche P800 Modular Cobas 8000; Roche).

### Analysis

Data are reported as mean ± SE. The primary outcome in this exploratory study was the association between UA levels and BP at baseline and in response to Ang‐II infusion at 60 min as a measure of RAS activity, stratified by sex. Secondary outcomes were the association between UA levels and measures of arterial stiffness at baseline and in response to Ang‐II infusion. Associations between UA concentrations BP, and arterial stiffness, at baseline and in response to Ang‐II challenge were analyzed by univariate analysis. Multiple linear regression analyses were employed to evaluate these associations further adjusting for covariates. The following variables were included in the models in addition to UA level: age, sex, body mass index (BMI), urine albumin excretion (UAE). Mean arterial pressure (MAP) was also included in the model for analysis of association between UA and measures of arterial stiffness. Baseline values of SBP, DBP, PWV, and AIx were included in the analysis of their respective responses to Ang‐II infusion, respectively. Wilcoxon signed‐rank test was used to compare values at 60 min with baseline values for each outcome variable. Sex comparisons were conducted with either an unpaired *t*‐test or the Mann–Whitney *U*‐test. All model assumptions were tested and met. All statistical analyses were performed with the statistical software package SPSS V.19.0 (SPSS, Chicago, IL) and were two‐tailed with a significance level of 0.05. Due to concerns regarding multiple comparisons and thus increasing the chance of a Type 1 error by detecting a difference by random chance alone, we a priori elected to compare the responses between men and women at the end of the AngII infusion.

## Results

### Baseline characteristics

Subject characteristics are presented in Table 1. Subjects were healthy, normotensive, nonobese, nondiabetic, and in high‐salt balance. The majority of subjects were Caucasian and approximately one‐third were men. Men had a higher BMI compared to women, but this difference was not significant (*P* = 0.2). UA levels were significantly higher in men compared to women (*P* < 0.001) but both were within the normal sex‐specific range (Calgary‐Lab‐Services). Men had significantly lower urinary albumin than women (*P* = 0.046) but all values were within normal range (<30 mg/day) as defined by KDIGO guidelines (KDIGO 2013).

**Table 1.** Baseline characteristics.

Parameter	All subjects	Men	Women
*N*	52	17	35
Age (year)	39 ± 2	41 ± 4	38 ± 2
BMI (kg/m^2^)	26 ± 1	27 ± 1	25 ± 1
Race *Caucasian* (%)	81	100	71
Gender *Female* (%)	67	N/A	N/A
Serum Uric acid (μmol/L) (normal range)	272 ± 9	328 ± 14 (210–490)	247 ± 10* (140–350)
Glucose (mmol/L)	4.6 ± 0.07	4.7 ± 0.1	4.5 ± 0.09
Serum Cholesterol (mmol/L)	4.2 ± 0.1	4.2 ± 0.2	4.1 ± 0.1
24‐hour Urine Sodium (mmol/day)	240 ± 36	219 ± 37	261 ± 67
Urine Albumin (mg/day)	5 ± 1	3 ± 0.4	5 ± 1*
Plasma renin activity (ng/L/sec)	0.2 ± 0.02	0.3 ± 0.04	0.2 ± 0.02*
Aldosterone (ng/dL)	129 ± 11	164 ± 22	111 ± 11*
Angiotensin II (pmol/L)	20 ± 2	19 ± 2	20 ± 3

Values are mean ± SE. BMI, body mass index.

* *P* < 0.05 vs. men.

### UA and BP

#### Baseline measures

Baseline BPs were within normal range for both men and women but were significantly higher in men (SBP: *P* = 0.005; DBP: *P* = 0.05) (Table 2). When all subjects were analyzed together, there was a significant positive association between UA and baseline SBP (*r* = 0.54, *P* < 0.001), even after adjustment for covariates (*P* = 0.023). A positive association between UA and baseline DBP (*r* = 0.45, *P* = 0.001) was also observed but this association did not achieve significance after multivariable analysis (*P* = 0.1).

**Table 2.** Responses to ANG‐II challenge.

Parameter		SBP*	DBP*	PWV*	AIx*
All subjects	Baseline	115 ± 2	68 ± 1	7.7 ± 1.4	11.1 ± 2.2
	30 min	128 ± 3	79 ± 1	N/A	N/A
	60 min	135 ± 3†	81 ± 1†	9.0 ± 1.5†	23.2 ± 2.0†
Men	Baseline	122 ± 4	71 ± 2	8.3 ± 0.3	3.6 ± 4.4
	30 min	136 ± 5†	83 ± 2†	N/A	N/A
	60 min	141 ± 6†	83 ± 3†	9.7 ± 0.3†	17.0 ± 4.0†
Women	Baseline	111 ± 2‡	66 ± 1	7.3 ± 0.2‡	14.8 ± 2.3‡
	30 min	124 ± 3†‡	77 ± 1†‡	N/A	N/A
	60 min	132 ± 3†	80 ± 1†	8.6 ± 0.3†‡	25.8 ± 2.2^v^

Values are means ± SE. SBP, systolic blood pressure; DBP, diastolic blood pressure; PWV, pulse‐wave velocity; AIx, aortic augmentation index; N/A, not available.

* Means of two readings.

† *P* < 0.05 vs. baseline.

‡ *P* < 0.05 vs. same time‐point in men.

**Figure 1.**
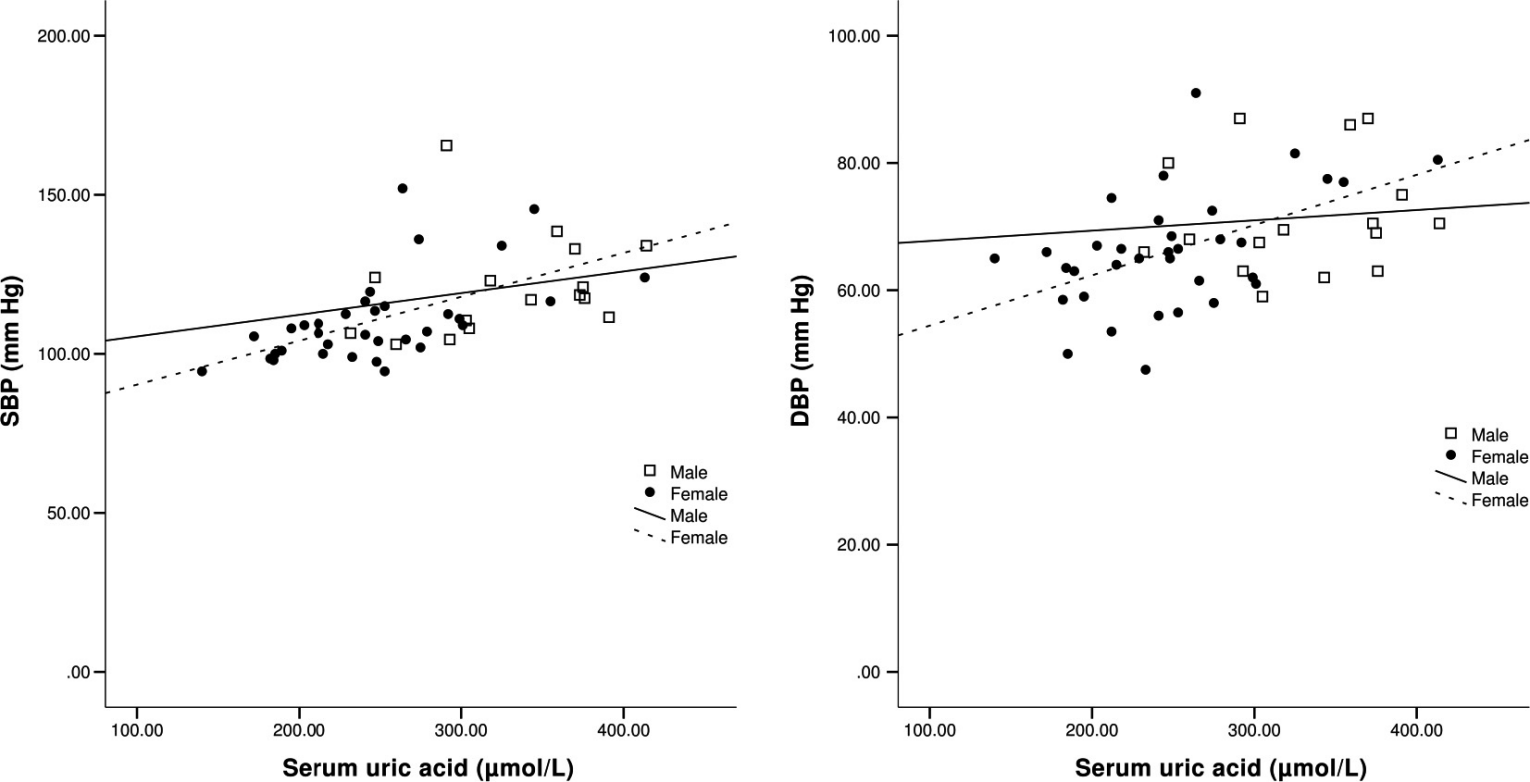
BP at baseline as a function of UA. Abbreviations: SBP, systolic blood pressure; DBP, diastolic blood pressure.

When the subjects were analyzed by sex, striking differences between men and women became apparent. In men, no association was observed between UA and BP on either univariate (SBP, *P* = 0.4; DBP, *P* = 0.7) (Fig. 1) or multivariable analysis (SBP, *P* = 0.7; DBP, *P* = 0.2). In stark contrast, women demonstrated a significant association between UA and baseline SBP with univariate (*r* = 0.57, *P* < 0.001) (Fig. 1) and multivariate analysis (*P* = 0.005). A similar relationship was observed between UA and DBP (univariate *r* = 0.49, *P* = 0.003 (Fig. 1), multivariate *P* = 0.02).

There is no absolute cut‐off for a pathologically elevated uric acid level. Although hyperuricemia is variably defined as a serum urate level greater than either 404 or 416 μmol/L (Terkeltaub 2010; Neogi 2011), there is no consensus as to when to institute pharmacological therapy in the absence of symptoms. However, as guidelines recommend the goal of maintaining UA levels below 300 μmol/L to prevent gout and its debilitating consequences (Jordan et al. 2007; Khanna et al. 2012), we chose 300 μmol/L as a conservative cut‐off to stratify the study subjects into the “high” and “low” uric acid groups. As such, all subjects were stratified into “low‐UA” (UA ≤300 μmol/L) or “high‐UA” (UA >300 μmol/L) groups. The low‐UA group (UA level 236 ± 7 μmol/L, men, *n* = 6, women, *n* = 30) demonstrated a significant association between UA level and BP (SBP, *r* = 0.45, *P* = 0.008; DBP, *r* = 0.31, *P* = 0.08), an association that was not observed in the high‐UA group (UA level 354 ± 9 μmol/L, men, *n* = 11, women, *n* = 5) (SBP, *P* = 0.242, DBP, *P* = 0.138). Sensitivity analyses using only the carotid‐femoral PWV data or only the carotid‐radial PWV data did not show an association between uric acid level and this measure of arterial stiffness.

#### Response to Ang‐II challenge

As anticipated, all subjects demonstrated significant increases in all indices of BP and arterial stiffness (Table 2).

**Figure 2.**
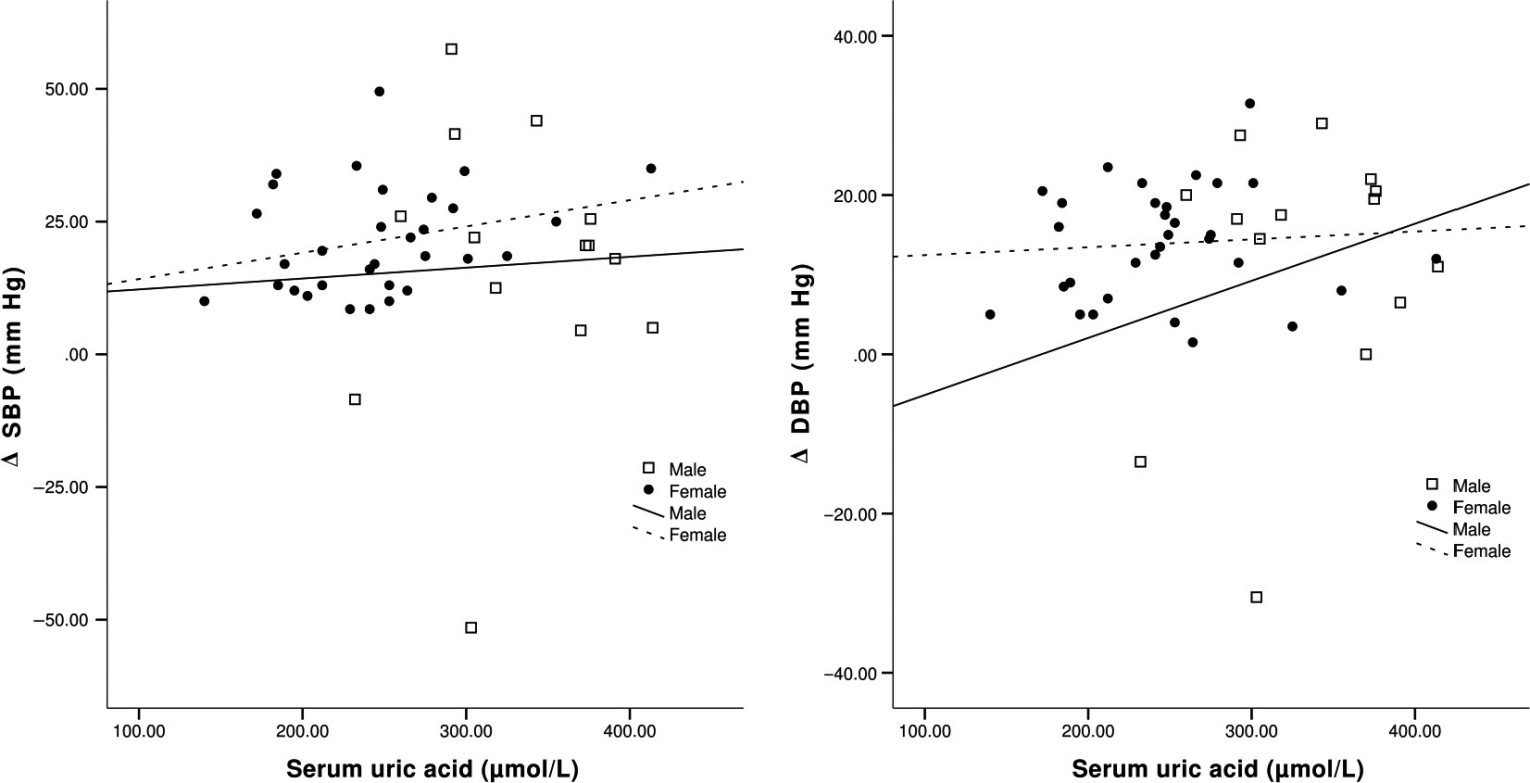
BP response to Ang‐II as a function of UA. Abbreviations: ΔSBP, change in systolic blood pressure; ΔDBP, change in diastolic blood pressure.

When analyzing all individuals together, there was no association between UA and the change in SBP (*P* = 0.8) or DBP (*P* = 0.7) in response to Ang‐II infusion, even after adjustment for covariates. When stratified by sex, men did not show any association between UA levels and the BP response to Ang‐II (Fig. 2). In contrast, women demonstrated a greater increase in SBP *P* = 0.035) and DBP (*P* = 0.056) with increased UA levels after adjustment for covariates (Fig. 2). Stratifying subjects into low‐ and high‐UA groups demonstrated a trend toward an association between UA level and the BP response to Ang‐II challenge in the low‐UA group (SBP: *r* = 0.35; *P* = 0.051; DBP; *r* = 0.34, *P* = 0.06), but not in the high‐UA group (SBP, *P* = 0.27, DBP, *P* = 0.49).

#### UA and arterial stiffness

When all subjects were analyzed together, a positive relationship was observed between UA and baseline PWV (*r* = 0.30, *P* = 0.04) but this relationship lost significance when adjusted for covariates (*P* = 0.9). However, a significant association was observed between UA and baseline AIx after adjustment for covariates (*P* = 0.05). Stratification by sex did not reveal any significant associations between UA and baseline measures of arterial stiffness. Similarly, stratification according to low‐ and high‐UA status (≤300 μmol/L and >300 μmol/L and) did not reveal any significant associations between UA and baseline measures of arterial stiffness. No association was observed between UA and the arterial stiffness response to Ang‐II, even after stratification by sex or UA group status.

## Discussion

This is the first study to examine the impact of sex on the relationship between UA and arterial tone at baseline and in response to Ang‐II challenge in young, healthy humans. Our key findings were as follows: (1) women demonstrated an association between UA concentrations and baseline BP, but men did not; (2) this apparent sex‐related difference was attributable to disparities in baseline UA levels rather than sex‐mediated pathophysiology, (3) UA level was associated with arterial stiffness, but this relationship did not differ by sex or UA group status. The dichotomy in the association between UA and BP observed between our healthy men and women, both at baseline and in response to Ang‐II supports the “two phase” hypothesis proposed by Feig et al. (2013), whereby initially high‐UA levels result in vascular dysfunction but after exposure to chronic elevation of UA, changes in UA level do not result in any corresponding alterations in the vasculature. The results of this study thus suggest that the stronger associations observed between UA and cardiovascular outcomes in women may simply be a reflection of the fact that women have not been exposed to persistently elevated UA levels. As such, the female vasculature may remain more sensitive to alterations in UA level.

Previous studies have suggested a “threshold effect” for the impact of UA on cardiorenal outcomes, suggesting that UA might be either a protective or a risk factor, when, respectively, present at normal or increased concentrations (Lippi et al. 2008). Others have shown increased UA concentrations to be a greater risk factor in women compared to men (Kivity et al. 2013a,b) despite women consistently having lower concentrations of this purine metabolite. Feig and colleagues have proposed that hypertension develops in two stages, whereby initially salt‐resistant blood pressure can be reduced by pharmacologically decreasing uric acid levels (Feig et al. 2013). Subsequently, chronically elevated blood pressure becomes increasingly salt‐sensitive and dependent on renal microvascular disease and low‐grade renal inflammation associated with persistent renal vasoconstriction and renal ischemia (Rodriguez‐Iturbe et al. 2007). At this point, lowering of uric acid levels no longer results in any significant reduction in blood pressure. Experimental studies suggest that uric acid might have a role in initiating the development of hypertension and that over time the importance of the serum uric acid would shift more to the role of the kidney in maintaining a salt‐sensitive hypertensive state rather than contributing directly to vascular pathology. We postulate that all of our subjects were salt‐resistant given they remained normotensive despite the high‐salt diet. This hypothesis may help explain discrepancies between studies examining the association between UA and BP (Forman et al. 2007; Feig et al. 2008; Soletsky and Feig 2012; Dawson et al. 2013). In support of this theory, limited animal studies have suggested a role for UA as a causative agent in increasing BP (Mazzali et al. 2001, 2002; Sanchez‐Lozada et al. 2002, 2005). Mazzali et al. (2001) demonstrated that the inhibition of the UA oxidizer, uricase, in rats led to an increase in UA to levels comparable to that observed in humans. This resulted in a significant increase in BP. However, the absence of uricase in humans limits the applicability of these animal studies, underscoring the importance of human studies. In keeping with the “two phase” hypothesis, the association between UA and BP was shown to be significant in adolescents (Feig et al. 2008; Soletsky and Feig 2012). In a randomized, placebo‐controlled study (Feig et al. 2008), allopurinol, a xanthine oxidase inhibitor that lowers UA levels, significantly reduced BP compared to placebo.

Several studies have demonstrated age and sex‐related variations in metabolism of UA (Mikkelsen et al. 1965; Scott and Pallard 1970) thereby resulting in a range of UA concentrations. It has been demonstrated that men show an increase in UA concentrations after the onset of puberty where it reaches a plateau and its levels remain stable afterward (Wolfson et al. 1949; Kelley 1981). However, women in the postpubertal and premenopausal stage show a lower level of UA in relation to men of similar ages (Mikkelsen et al. 1965) but their UA levels reach that of men during the postmenopausal stage (Kelley 1981). Possible explanations for these variations are higher renal excretion of UA and lower UA synthesis in women (Anton et al. 1986). Animals studies have demonstrated that there are two stages to developing hypertension: an initial intravascular stage, where hypertension can be averted by lowering UA levels with allopurinol or increasing excretion of UA using uricosuric agents (Mazzali et al. 2001; Sanchez‐Lozada et al. 2008), and the chronic intracellular stage, where hypertension is kidney dependent and becomes independent of UA levels; hypertension persists despite lowering UA level to the normal levels (Watanabe et al. 2002). Similar results have been supported by clinical studies (Johnson et al. 2002; Rodriguez‐Iturbe et al. 2007). Thus, it is possible that due to chronically higher levels of UA, men transition at an earlier stage from the initial intravascular stage into the intracellular stage and BP is therefore becoming independent of and insensitive to UA levels; as such any increase in UA levels would have no effect on BP. However, due to the consistently lower levels of UA observed in women, it is expected that in females the vasculature remains in the initial intravascular, UA‐dependent stage. This suggests that any increase in UA levels in women would translate into an increased BP and thus, poorer cardiovascular outcomes, whereas the same increase in UA would have not have this same effect in men.

Our findings of an association between UA and AIx but not PWV are similar to those of Naqvi et al. (2013) and Cicero et al. (2014), respectively. In another study, arterial stiffness, evaluated by PWV, was positively associated with UA levels in postmenopausal women (Park et al. 2012). Similarly, UA was positively correlated with PWV in hypertensive patients with a mean age of 62 years (Gómez‐Marcos et al. 2013). In another study, UA, within its normal range, was shown to be positively correlated with arterial stiffness, as measured by PWV_ba_ (brachial‐ankle PWV) in Korean men but not women (Shin et al. 2012). Discrepancies between these studies and the present one may reflect the differences in populations, hypertensive and other comorbidity status, age and study conditions, as our study population comprised of normotensive subjects in a high‐salt state and variances in menstrual cycle were controlled for by studying premenopausal women. Furthermore, the hypothesis regarding the relationship between uric acid and the vasculature proposed by Feig and colleagues is in the context of hypertension and blood pressure control, with a particular focus on the adolescent and young adult population free from comorbidities. The lack of association between uric acid levels and measures of arterial stiffness in our study may simply reflect different vascular beds.

There are significant differences in arterial stiffness between men and women, with greater arterial stiffness described in the women compared with age‐matched men (Hayward and Kelly 1997). In healthy women, serum uric acid levels >273 μmol/L or greater were associated with greater risk of arterial stiffness, though the relationship between hyperuricemia and increased arterial stiffness was not significant in men (Fang et al. 2014). This suggests that sex differences in arterial stiffness may at least be partially explained by differences in baseline uric acid.

Previous studies have suggested a link between UA and renin–angiotensin system activity. Perlstein et al. (2004) demonstrated a positive association between UA and the renal RAS using a protocol similar to that employed in this study. UA stimulates Ang‐II production in vascular smooth muscle cells, suggesting that UA causes cardiovascular disorders by stimulating the vascular RAS (Corry et al. 2008). A more sensitive vascular response to Ang‐II has been interpreted as consistent with down‐regulated RAS activity (Shoback et al. 1983a; Price et al. 2002; Ahmed et al. 2004; Miller et al. 2006; Cherney et al. 2008; Forman et al. 2010; Vaidya et al. 2012; Nicholl et al. 2014; Zalucky et al. 2014). Our findings of an association between increased uric acid levels and an increasingly vasoconstrictor response to AngII in women are therefore a reflection of lower local tissue–AngII concentrations and tissue–RAS activity. As such, the increasingly greater vasoconstrictor response to Ang‐II with higher levels of UA in women may be interpreted as protective and may reflect the purported antioxidant effects of UA (Johnson et al. 2013). Uric acid administration substantially increases in vivo antioxidant capacity in healthy volunteers with low baseline serum concentrations, consistent with normal antioxidant defenses and low oxidant stress (Waring et al. 2001). However, as Ang‐II also induces xanthine oxidase activation in endothelial cells (Landmesser et al. 2007), it is possible that the higher uric acid observed in the women with the greatest vasoconstrictor response is simply a consequence of lower vascular AngII levels decreasing xanthine oxidase activation with the end result of greater uric acid levels.

Uric acid levels vary across the menstrual cycle and show associations with endogenous estradiol (E2) and reproductive hormone concentrations in regularly menstruating women. Mumford et al. (2013) reported the highest uric acid concentrations during the follicular (low E2) phase. Furthermore, uric acid levels were inversely associated with E2 and progesterone, and positively associated with follicular stimulating hormone. In keeping with the concept that estradiol influences uric acid concentrations, Wingrove et al. (1998) reported significantly higher uric acid concentrations in postmenopausal versus premenopausal women, consistent with lower estrogen levels among postmenopausal women although whether this was accompanied by a corresponding decrease in blood pressure remains unknown.

Estradiol (E2), both endogenous and exogenous has been suggested to lower the uric acid level through mechanisms involving renal clearance, secretion, and reabsorption (Nicholls et al. 1973; Hak and Choi 2008; Mumford et al. 2013). While the role of ovarian sex hormones on uric acid production, metabolism, and excretion is not well understood, estradiol appears to decrease uric acid levels by lowering the postsecretory tubular reabsorption of uric acid in the nephron and thus decreasing increasing the fractional excretion of uric acid (Puig et al. 1986; Yahyaoui et al. 2008), which may account for intersex differences in uric acid levels. However, administration of exogenous estradiol does not consistently have the same effect (Adamopoulos et al. 1977; Anton et al. 1986; Puig et al. 1986; Sumino et al. 1999). As such, further studies should elucidate these hypotheses more directly in both men and women.

This study has limitations. The study subjects were healthy, normotensive, and nonsmoking and not reflective of the general population, thus limiting generalizability. However, studying a healthy population allowed us to minimize the effect of confounding factors in determining the association between UA and measures of BP and arterial stiffness. We also controlled for effects of sex hormones on vascular measures and the RAS (Chidambaram et al. 2002; Ahmed et al. 2004) by studying women in the same phase of menstrual cycle (premenopausal) and by studying women who were not ingesting oral contraceptives. Because salt intake is a major determinant of not only the state of the RAS but also the contribution of renin activity to vascular tone, in this study we undertook to assess the vascular response to exogenously administered Ang‐II in the high‐salt state when endogenous Ang‐II levels are lowest. As such, we ensured that all subjects were in high‐salt balance, a state of maximal RAS suppression (Shoback et al. 1983b) thus allowing for meaningful comparisons across groups. Moreover, diet and time of blood sampling have been shown to influence UA levels in healthy populations (Plumelle et al. 2014). As such, we examined the association between UA and vascular parameters in a carefully controlled setting in the fasting state, thus optimizing study conditions. Our study population was almost exclusively Caucasian, which may limit the generalizability of our results to other racial populations where vascular dysfunction is more prevalent, such as those of African‐American or Aboriginal descent. A previous study did not detect an effect of race on the association between uric acid and the renovascular response to AngII challenge in a population of healthy normotensive and hypertensive subjects (Perlstein et al. 2004). Given the low proportion of non‐Caucasian subjects in this and our studies; however, it is still possible that race may still be a modifier in the effect of uric acid on vascular tone. Finally, due to the cross‐sectional nature of our study design, we cannot demonstrate directionality of the association nor comment on causality and our results are hypothesis‐generating rather than definitive proof. Nevertheless, our study suggests that in the healthy population, sex differences in the association between UA and vascular outcomes may be attributable to the fact that women demonstrate lower levels of UA compared to men rather than sex‐mediated differences in physiology.

## Perspectives

In our healthy, normotensive population with serum UA levels within the normal range, we found that increased levels of UA were associated with both increased baseline BP and Ang‐II responsiveness in women but not in men. This apparent sex difference appeared to be due to the lower UA levels observed in women compared to men rather than pathophysiological sex‐mediated differences.

## Conflict of Interest

There are no conflicts of interest.
